# Transnasal Esophagorumenoscopy and Laparoscopic Assisted Rumenostomy in a Cow With Digestive Distress—Case Report

**DOI:** 10.1155/crve/8183318

**Published:** 2026-03-09

**Authors:** Luiz Henrique Vilela Araújo, Luis Gustavo e Silva Novais, Thiago da Silva Cardoso, Lucas Santos Carvalho, Pedro Henrique Lira Cerqueira, José Leandro da Silva Gonçalves, André de Medeiros Costa Lins, Tatiane Teles Albernaz Ferreira, José Alcides da Silveira, Marco Duarte Dutra, Felipe Masiero Salvarani, Francisco Décio de Oliveira Monteiro, Pedro Paulo Maia Teixeira

**Affiliations:** ^1^ Veterinary Medicine Institute, Federal University of Pará, Castanhal, Pará, Brazil, ufpa.br; ^2^ Federal Institute of Tocantins (IFTO), Campus Araguatins, Araguatins, Tocantins, Brazil

**Keywords:** diffuse peritonitis, esophageal obstruction, ruminal impaction, transfaunation, veterinary endoscopy

## Abstract

A Girolando cow presented with signs suggestive of esophageal obstruction. The team performed diagnostic and therapeutic procedures. Transnasal esophagorumenoscopy with a flexible gastroscope revealed hyperemic erosions and later foamy ulcers in the esophageal mucosa, but no obstructive foreign body. Laparoscopic‐assisted rumenostomy via a two‐port technique identified intraperitoneal adhesions and ruminal impaction. Under laparoscopic guidance, we placed and secured a handmade cannula fashioned from a 10‐mm cuffed endotracheal tube to the abdominal wall. This provided access for therapeutic transfaunation and fluid administration, facilitating short‐term clinical stabilization. However, the cow ultimately died due to complications from traumatic reticuloperitonitis caused by a perforating metallic foreign body, leading to diffuse peritonitis and aspiration pneumonia, as confirmed by necropsy. The primary sequelae were diffuse peritonitis and aspiration pneumonia, the latter likely secondary to the esophageal dysfunction and/or therapeutic intervention, as confirmed by necropsy. Despite the fatal outcome, transnasal endoscopy offered valuable real‐time assessment of esophageal integrity, and the laparoscopic‐assisted placement of a ruminal cannula proved to be a functional, minimally invasive therapeutic alternative. This case illustrates the combined utility of these endoscopic techniques for diagnosing and managing complex digestive distress in cattle, while highlighting their limitations in reversing severe, established pathology.

## 1. Introduction

Transnasal endoscopy and exploratory laparoscopy are valuable diagnostic tools to address digestive distress in ruminants. Transnasal esophagorumenoscopy allows for detailed visualization of the esophageal mucosa and, when visibility is not impeded by contents, portions of the proximal rumen. The procedure is capable of supporting diagnoses of hyperemic erosions or ulcers in the esophagus and can reveal alterations in ruminal fluid character and accessible mucosal surfaces, in addition to providing a safe collection of samples from biopsies, thus being a well‐tolerated and minimally invasive procedure [1–3]. In addition, laparoscopy is an innovative technique applicable in field conditions, providing detailed and enhanced visualization of intraperitoneal organs and structures [4, 5]. These minimally invasive approaches facilitate precise inspection of organs and tissues, offering a clear, amplified, and vivid view [2]. The diagnostic importance of these techniques lies in the endoscopic findings themselves and the ability to perform biopsy, puncture sampling, or lavage under endoscopic guidance, ensuring safe interventions and accurate diagnoses [5].

Exploratory laparoscopy through the paralumbar fossa is a highly valuable complementary method to diagnose intra‐abdominal distress in cattle [6]. Laparoscopy enables the assessment of bowel motility and is crucial to differentiating between primary and secondary digestive disorders, thus improving both diagnosis and treatment. The use of these minimally invasive approaches results in targeted treatments while reducing the risk of postoperative complications [7].

Rumenostomy is a widely used procedure for both digestion research and therapeutic purposes [8]. It can be performed using standard open technique or mini‐invasive approaches, such as the laparoscopic assisted method. Endoscopy guided rumenostomy is a feasible technique, characterized by low complexity and reduced complications [9]. Furthermore, laparoscopic assisted ruminal cannulation is a promising approach, providing safe and effective fluid delivery directly to the rumen [10, 11].

Digestive distress in cattle can arise from various causes, often requiring advanced diagnostics for accurate management. A common example is esophageal obstruction, typically due to foreign body ingestion, which not only causes primary digestive dysfunction but can also lead to severe secondary complications like aspiration pneumonia [12–14]. Such respiratory complications are frequently associated with esophageal disorders and can be evaluated through clinical and ultrasonographic assessment [15, 16]. Another serious condition is traumatic reticuloperitonitis, which carries a high‐mortality rate and for which minimally invasive diagnostic and therapeutic approaches are proving increasingly valuable [17]. The diversity and severity of digestive diseases, particularly in challenging environments like semiarid regions, underscore the general need for sophisticated diagnostic tools in bovine practice [18].

This case report highlights the application of transnasal esophagorumenoscopy and laparoscopic‐assisted rumenostomy for the treatment of digestive disorders in a cow.

## 2. Case

A 7‐year‐old Girolando cow (430 kg) was admitted (Day 0) to the veterinary teaching hospital with a 2‐day history of dyspnea, left abdominal distension, and sialorrhea. Before referral, the owner had attempted to relieve a suspected esophageal obstruction by passing a tube orally. The diagnostic and therapeutic timeline proceeded as follows: initial examination and treatment on Day 0; first transnasal endoscopy on Day 1; second transnasal endoscopy on Day 7; laparoscopic‐assisted rumenostomy on Day 8; and exploratory laparotomy, euthanasia, and necropsy on Day 14.

The management of this clinical case was conducted in accordance with the standard clinical care protocols and animal welfare guidelines of the Veterinary Teaching Hospital, Federal University of Pará. Informed written consent was obtained from the owner for all described procedures, including endoscopic examinations, surgical interventions, and euthanasia.

### 2.1. Clinical Findings

On initial physical examination (Day 0), the patient exhibited depression, anorexia, dyspnea, coughing, and a putrid exhaled odor. Vital signs included an increased respiratory rate (33 breaths per minute), increased heart rate (76 bpm) with arrhythmia, and a rectal temperature of 40°C. The rumen was tympanic and atonic.

Initial management included the administration of a combination of antibiotics (procaine benzylpenicillin and dihydrostreptomycin sulfate) at a dose of 20,000 IU/kg of the penicillin component (IM, SID, for 7 days) and flunixin meglumine (1.4 mg/kg, IM, SID, for 7 days). Vitamins B1 (thiamine, 2 mg/kg, IM, SID, for 2 days) and B12 (cyanocobalamin, 5 mL, IM, SID, for 2 days) were also administered.

### 2.2. Diagnostic Procedures

Medical management, including antibiotics, anti‐inflammatories, and vitamin supplementation, was initiated on Day 0. The first diagnostic procedure, a transnasal esophagorumenoscopy, was performed on Day 1 using a flexible endoscope (Endovision GDI; 1500 mm length, 9.8 mm diameter). With the cow in a standing position, the endoscope was inserted through the right nostril. The examination revealed hyperemic spots and severe fluid accumulation in the distal esophagus, with no obstructive foreign body identified.

Due to persistent clinical signs, a second transnasal endoscopy was performed on Day 7. This examination showed progression of the esophageal lesions to ulcers covered with foam. The patient also exhibited a putrid exhaled odor and abundant nasal discharge during this procedure.

Given the ongoing ruminal atony and distension, a laparoscopic‐assisted rumenostomy was performed on Day 8. The left paralumbar fossa was clipped and aseptically prepared. The cow received mild sedation with xylazine hydrochloride (0.05 mg/kg, IV) followed by local anesthesia of the paralumbar fossa using an inverted “L” block with 2% lidocaine chloride. A digital rigid endoscope (10 mm diameter, 325 mm length) connected to a mobile device was used. A two‐port technique was employed: The first (camera) port was established using a modified Hasson technique, revealing moderate intraperitoneal adhesions between the rumen and abdominal wall. A second port was then inserted into the rumen under laparoscopic guidance.

The telescope was transitioned to this second port, providing an intraruminal view of impacted content and atrophied papillae. Prior to its reintroduction into the abdominal cavity, the telescope was meticulously cleansed and disinfected to prevent iatrogenic contamination. Subsequently, under continuous laparoscopic visualization, a handmade cannula, fashioned from a 10‐mm cuffed tracheal tube, was introduced through the second port, advanced into the rumen, and secured to the abdominal wall muscles with suture. The cuff was inflated to seal the cannula within the rumen. The total duration of this procedure was approximately 33 min.

### 2.3. Treatment

The laparoscopic‐assisted rumenostomy procedure was completed without complications. Several cutaneous scars were noted on the paralumbar fossa, possibly from previous percutaneous ruminal interventions. Postoperative care following the rumenostomy (Day 8) included the administration of benzathine benzylpenicillin (20,000 IU/kg, IM, SID) and flunixin meglumine (2.2 mg/kg, IM, SID), both continued for 3 days. As part of the supportive therapy, therapeutic transfaunation using 6 L of ruminal fluid from healthy cattle alongside 10 L of water was administered daily through the ruminal cannula for the first 3 postoperative days. Further fluid administration was subsequently hindered by the rumen becoming completely filled.

### 2.4. Outcome

The patient was monitored intensively twice daily for 9 days following the rumenostomy (Days 8–14). During the first 5 days, clinical parameters were stable: The cow remained active with normal mucous membranes, a capillary refill time of 2 s, and a mean ruminal movement count of 2–3 incomplete movements per minute. Vital signs included a mean heart rate of 72 bpm, a respiratory rate of 28 breaths per minute, and a core temperature of 38.4°C. Feces were diarrheic, whereas urine was normal.

**Figure 1:**
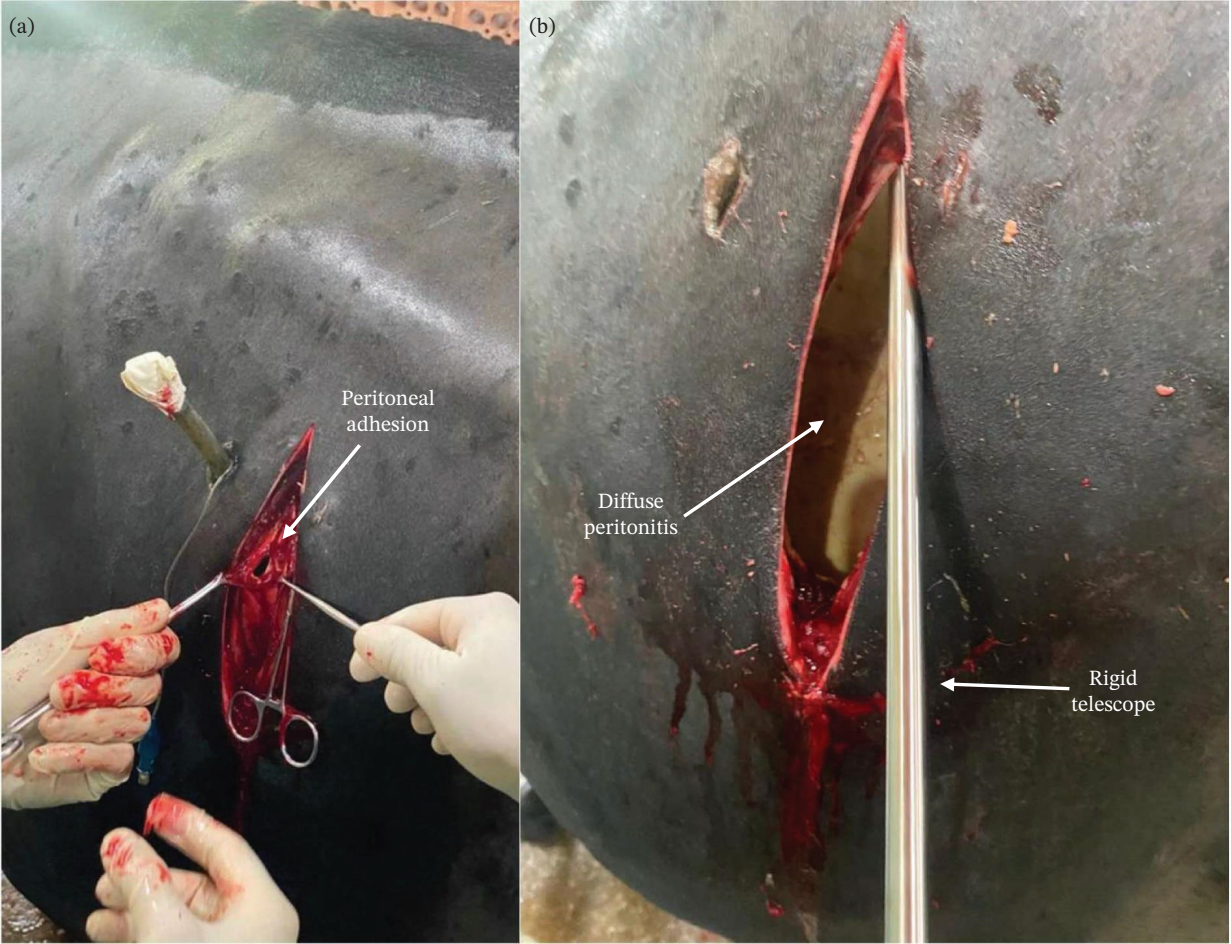
Exploratory laparotomy in a cow after 9 days of laparoscopic assisted rumenostomy. (a) Adhesions (arrow) at the peritoneal incision site; (b) use of the digital rigid telescope for intra‐abdominal view during laparotomy, revealing diffuse fibrinous peritonitis.

However, on Day 14 (6 days postrumenostomy), the patient′s status deteriorated acutely, presenting with depression, anorexia, and cessation of ruminal movements. Due to this decline, an exploratory laparotomy was performed on the same day (Figure 1a). The surgical exploration revealed severe diffuse fibrinous peritonitis and extensive adhesions between the rumen, reticulum, and abdominal wall (Figure 1a,b).

The cow was restrained in a standing position and the left paralumbar fossa was prepared and anaesthetized aseptically as previously described. A 15‐cm vertical laparotomy was performed on the left flank, starting slightly caudal to the ruminal cannula. The paralumbar fossa muscles were bluntly dissected, followed by a sharp incision of the peritoneum with standard scissors (Figure 1a). Using the same digital rigid endoscope previously reported, it was inserted through the laparotomy incision without the need for insufflation, providing a magnified and illuminated view of the deeper intraperitoneal structures. Endoscopic imaging revealed severe diffuse peritonitis on the left side of the peritoneal cavity (Figure 1b). Percutaneous rumenoscopy revealed signs of regeneration of the mucosal papilla and impacted material within the rumen. Through abdominal endoscopic inspection, severe intraperitoneal adhesions were observed between the rumen, reticulum, and abdominal wall. Abdominal wall closure was performed using cruciate (cross) sutures for both muscular layers and skin, with 2‐USP nylon sutures.

Given the grave prognosis confirmed by these findings, euthanasia was performed immediately, followed by necropsy. The decision for euthanasia was based on the progression of irreversible clinical deterioration, specifically: persistent anorexia, complete absence of ruminal motility, confirmation of severe diffuse fibrinopurulent peritonitis with extensive adhesions via exploratory laparotomy, and a diagnosis of aspiration pneumonia, collectively indicating a grave prognosis.

**Figure 2:**
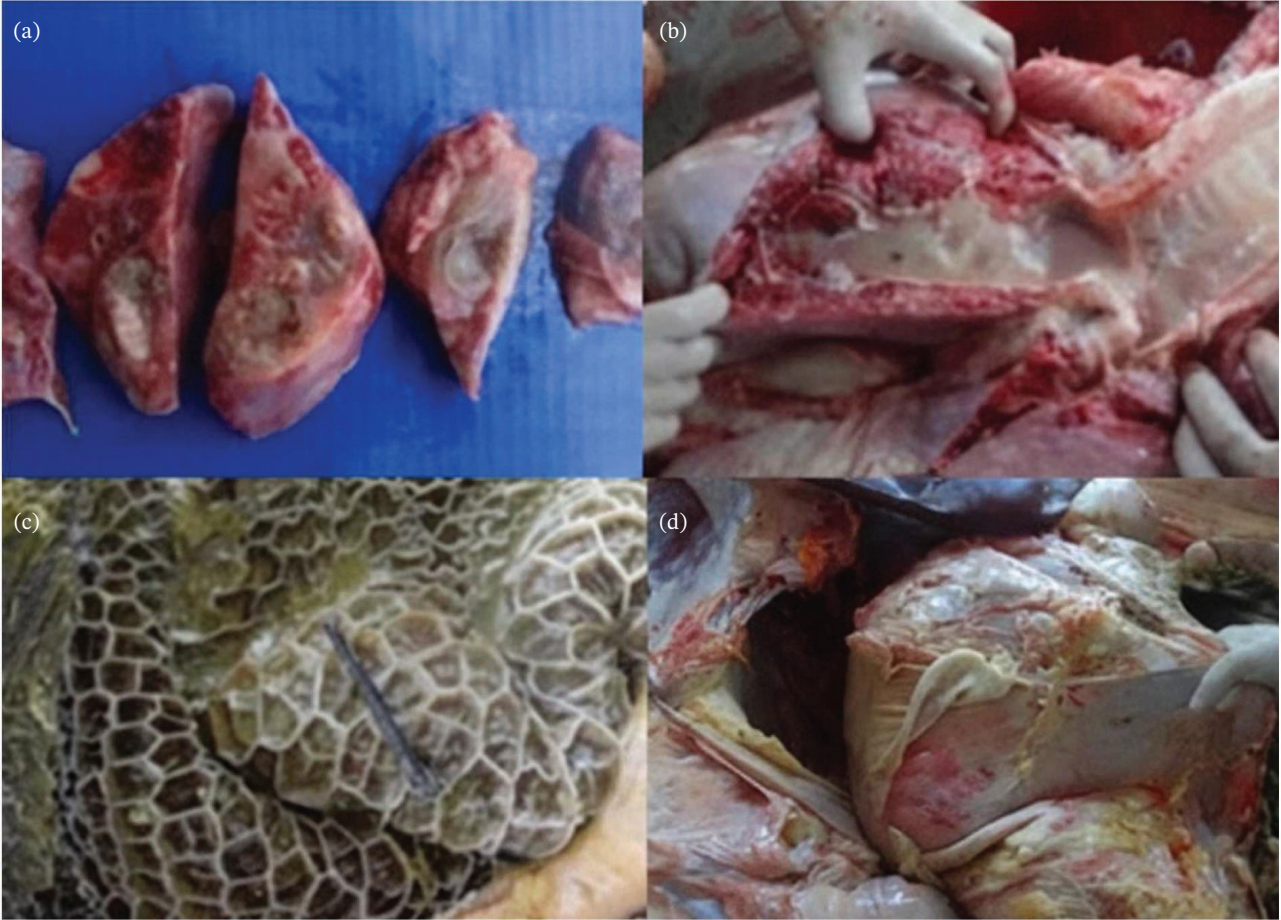
Macroscopic postmortem findings. (a) Multifocal abscesses (0.5–2 cm diameter) in the cranioventral left lung. (b) Foamy, edematous content within the right bronchus. (c) Metallic foreign body (arrow) recovered from the reticulum. (d) Extensive fibrin deposition and adhesions within the abdominal cavity.

The findings of necropsy of the cranioventral aspect of the abdomen revealed the presence of multifocal abscesses (Figure 2a), ranging from 0.5 to 2 cm in diameter, containing purulent material with a foul smell. The left lung contained a large amount of greenish purulent fluid, whereas the right lung was filled with a foamy substance that resembled pulmonary edoema (Figure 2b). In the abdominal cavity, there was extensive fibrin deposition near the pelvis, peritoneum, and laterally to the fermentation chambers. The abomasal mucosa presented multifocal ulcers. The rumen serosa exhibited multiple fibrinous adhesion bands (Figure 2c), and a metallic foreign body was found within the reticular content (Figure 2d). Thus, the final diagnosis was abomasal ulcers and reticuloperitonitis due to a penetrating reticular foreign body, diffuse peritonitis, and aspiration pneumonia. No histopathological analysis was performed.

The postmortem examination confirmed the final diagnosis of traumatic reticuloperitonitis caused by a perforating metallic foreign body, resulting in diffuse peritonitis, and, aspiration pneumonia secondary to the esophageal dysfunction and/or therapeutic intervention.

## 3. Discussion

The initial clinical presentation in this case (dyspnea, left abdominal distension, and sialorrhea) was suggestive of esophageal obstruction, a condition frequently associated with foreign body ingestion in cattle [12–14]. However, the endoscopic findings of hyperemia, erosions, and later ulcers were most consistent with iatrogenic mucosal trauma, likely resulting from the owner′s repeated attempts at oral intubation to relieve a presumed obstruction. The metallic foreign body identified during necropsy was located in the reticulum and was the cause of the ultimately fatal traumatic reticuloperitonitis (hardware disease), a distinct syndrome from esophageal obstruction [17]. The aspiration bronchopneumonia, confirmed postmortem, is a recognized complication of esophageal dysfunction [15, 16] and in this case was likely secondary to the esophageal trauma and associated dysphagia/sialorrhea.

Although the presumptive diagnosis of obstruction was made based on history and clinical signs, transnasal esophagorumenoscopy provided critical, case‐specific information that traditional physical examination could not. It directly visualized hyperemic erosions and later, foamy ulcers within the esophageal mucosa. This objective assessment ruled out a persistent obstructive foreign body at the time of examination and confirmed significant mucosal trauma, likely exacerbated by the owner′s prior attempts at relief. This finding directly influenced management by shifting the focus from foreign body retrieval to managing esophagitis, ruminal impaction, and secondary complications. Furthermore, the endoscopic view of the rumen revealed impacted content and atrophied papillae, providing immediate visual confirmation of ruminal dysfunction that complemented the clinical finding of atony. Endoscopic biopsy techniques, including oral and nasoruminal approaches, have been validated as safe and effective methods for rumen sampling [1–3].

The application of transnasal esophagorumenoscopy in an adult cow with complex digestive distress underscores its clinical utility beyond simple foreign body retrieval. As a minimally invasive procedure, it offered a definitive visualization of the mucosa, aligning with its reported advantages of safety and tolerability [5]. The laparoscopic‐assisted rumenostomy performed in this case served a dual purpose: It was both a diagnostic modality, allowing for the identification of intraperitoneal adhesions and ruminal impaction, and a therapeutic intervention by establishing access for transfaunation and fluid therapy. Laparoscopy is increasingly recognized as a valuable tool for diagnosing abdominal disorders in cattle and facilitating minimally invasive therapeutic procedures [6, 7].

The specific combination of these two minimally invasive techniques, transnasal esophagorumenoscopy for proximal tract evaluation and laparoscopic‐assisted placement of a handmade tracheal tube as a rumenostomy cannula, for the management of such a case has not, to our knowledge, been previously detailed in cattle. Although laparoscopic rumenostomy has been described in small ruminants and experimental models [9, 11], its reported use in cattle, particularly in conjunction with proactive esophageal endoscopy for comprehensive diagnostic work‐up, appears novel. Laparoscopic‐assisted techniques have been successfully applied in various bovine procedures, including umbilical resection and ruminal cannulation [10, 11]. The handmade cannula functioned effectively for 9 days, providing a safe, low‐cost, and secure access point for therapy, thereby avoiding the immediate need for a more invasive conventional rumenostomy [8]. However, the application of these combined techniques is not without challenges. These include the requirement for specialized endoscopic equipment and surgical training, potential limitations in visualizing forestomach compartments due to ruminal content in adult cattle, and the inherent risks of iatrogenic injury during scope or trocar insertion.

Despite these interventions, the patient succumbed to diffuse peritonitis and pneumonia. The exploratory laparotomy performed on the ninth postoperative day confirmed the grave prognosis by revealing severe fibrinous peritonitis, which led to the decision for euthanasia. Subsequent necropsy provided definitive postmortem confirmation of these findings, including the identification of a perforating metallic foreign body. Traumatic reticuloperitonitis, as ultimately diagnosed, carries a high‐mortality rate, and euthanasia is often a recommended outcome in advanced cases with diffuse abdominal involvement [17]. This outcome highlights that although minimally invasive techniques offer excellent diagnostic capability and can stabilize patients, they cannot always reverse underlying severe pathology, such as that caused by a perforating foreign body. The prevalence and severity of digestive diseases in cattle, particularly in certain regions, further underscore the challenges in managing such cases [18].

This case demonstrates that the combined use of transnasal esophagorumenoscopy and laparoscopic‐assisted rumenostomy provides a comprehensive, minimally invasive approach for diagnosing and managing complex digestive distress in cattle. The endoscopic evaluation offered superior mucosal assessment compared with traditional methods, directly informing clinical decisions, whereas the laparoscopic technique enabled both diagnosis and the establishment of therapeutic access. These approaches align with the evolving role of endoscopy and minimally invasive surgery in bovine medicine [4, 5].

## 4. Conclusions

This case demonstrates that transnasal esophagorumenoscopy provides valuable, real‐time assessment of esophageal and ruminal mucosa in cattle with complex digestive distress, enabling targeted management beyond physical examination alone. The integration of this endoscopic approach with laparoscopic‐assisted placement of a handmade rumenostomy cannula allowed for both diagnosis and therapeutic intervention in a minimally invasive manner. Although these techniques facilitated clinical stabilization and offered a functional, low‐cost alternative to conventional rumenostomy, they were unable to reverse the severe sequelae of a perforating foreign body, namely diffuse peritonitis and aspiration pneumonia. These findings support the growing role of endoscopy in bovine internal medicine as a precise diagnostic tool while underscoring that even advanced, minimally invasive approaches cannot always overcome irreversible underlying pathology.

## Author Contributions

Conceptualization, methodology, project administration, and supervision: Luiz Henrique Vilela Araújo, Luis Gustavo e Silva Novais, and Pedro Paulo Maia Teixeira. Data curation, formal analysis and investigation, and resources: Thiago da Silva Cardoso, Lucas Santos Carvalho, Pedro Henrique Lira Cerqueira, and Francisco Décio de Oliveira Monteiro. Visualization, writing—original draft, and writing—review and editing: José Leandro da Silva Gonçalves, André de Medeiros Costa Lins, Tatiane Teles Albernaz Ferreira, José Alcides da Silveira, Marco Duarte Dutra, Felipe Masiero Salvarani, Francisco Décio de Oliveira Monteiro, and Pedro Paulo Maia Teixeira.

## Funding

No funding was received for this manuscript.

## Conflicts of Interest

The authors declare no conflicts of interest.

## Data Availability

The data that support the findings of this study are available from the corresponding author upon reasonable request.
